# Feature Fusion and Metric Learning Network for Zero-Shot Sketch-Based Image Retrieval

**DOI:** 10.3390/e25030502

**Published:** 2023-03-14

**Authors:** Honggang Zhao, Mingyue Liu, Mingyong Li

**Affiliations:** 1 School of Computer and Information Science, Chongqing Normal University, Chongqing 401331, China; 2 Chongqing National Center for Applied Mathematics, Chongqing 401331, China

**Keywords:** sketch retrieval, ResNet-50, attention, metric learning, feature fusion, triplet loss

## Abstract

Zero-shot sketch-based image retrieval (ZS-SBIR) is an important computer vision problem. The image category in the test phase is a new category that was not visible in the training stage. Because sketches are extremely abstract, the commonly used backbone networks (such as VGG-16 and ResNet-50) cannot handle both sketches and photos. Semantic similarities between the same features in photos and sketches are difficult to reflect in deep models without textual assistance. To solve this problem, we propose a novel and effective feature embedding model called Attention Map Feature Fusion (AMFF). The AMFF model combines the excellent feature extraction capability of the ResNet-50 network with the excellent representation ability of the attention network. By processing the residuals of the ResNet-50 network, the attention map is finally obtained without introducing external semantic knowledge. Most previous approaches treat the ZS-SBIR problem as a classification problem, which ignores the huge domain gap between sketches and photos. This paper proposes an effective method to optimize the entire network, called domain-aware triplets (DAT). Domain feature discrimination and semantic feature embedding can be learned through DAT. In this paper, we also use the classification loss function to stabilize the training process to avoid getting trapped in a local optimum. Compared with the state-of-the-art methods, our method shows a superior performance. For example, on the Tu-berlin dataset, we achieved 61.2 + 1.2% Prec200. On the Sketchy_c100 dataset, we achieved 62.3 + 3.3% mAPall and 75.5 + 1.5% Prec100.

## 1. Introduction

The goal of Sketch-Based Image Retrieval (SBIR) is to locate the desired photo in the database, and hand-drawn sketches are used as queries. Potential users can search without using a photo query; it is easier to define pose or orientation by sketch than by textual description, which is really convenient. It is widely used in sketch-based scene search and e-commerce-related fields. Hand-drawn sketches can be easily drawn using touch-screen devices such as smartphones and iPads. For example, Brazilian researchers Leo Sampaio et al. [1] proposed a scene designer. Scene Designer is a novel method for searching and generating images using free-hand sketches of scene compositions, i.e., drawings that describe both the appearance and relative positions of objects. Taking the drawn sketch as a query, the retrieval system can return some relevant photos according to the user’s intention. In contrast to the typical image retrieval problem [2], querying sketches and retrieving photos in a sketch retrieval task use different domains. A more practical and realistic environment was used to introduce the Zero-Shot Sketch-Based Image Retrieval (ZS-SBIR) [3], where the category of the query sketch is unknown throughout training.

Both traditional VGG-16 [4] and ResNet-50 [5] have a strong ability to extract local and global structural features. In recent years, researchers have also applied models with good embedding abilities to sketch inspection, such as the Siamese CNN proposed by German researchers Laura et al. [6], which is trained to learn descriptors encoding local spatio-temporal structures between the two input image patches. However, for the ZS-SBIR task, the sketch is so abstract that sometimes even the human eye cannot distinguish it. Three main factors limit the performance of ZS-SBIR: (1) The sketch is too abstract and lacks texture features. Most current methods are not good at extracting fine-grained and abstract image features. (2) There is a huge domain gap between sketches and photos, and their feature distributions are very different. It is more difficult to train a joint embedding model. (3) Semantic similarities between the same features in photos and sketches are difficult to reflect in deep models without textual assistance. The most recent methods [7,8,9,10,11,12] use word embeddings extracted from language models [13] as supervised signals to guide feature fusion of sketches and photos. Existing approaches have focused on the feature fusion problem. Other studies [14,15,16,17,18] use adversarial networks (based on complex sub-networks) to smooth the domain gap. These efforts enhance the performance of this cross-domain retrieval work.

However, because sketches are extremely abstract, the commonly used backbone networks (such as VGG-16 [4] and ResNet-50 [5]) cannot handle both sketches and photos. Semantic similarities between the same features in photos and sketches are difficult to reflect in deep models. Therefore, a better feature extraction technique is required. We suggest an end-to-end module termed Attention Map Feature Fusion (AMFF) to be the backbone network for feature extraction, which is inspired by EGFF [19], ExFuse [20], and ATTENTION [21]. We use multi-layer features, considering both global and local features, and fuse them to produce rich features to better serve the ZS-SBIR objective. We fuse multi-layer features that employ a similar approach to EGFF and ExFuse, where EGFF employs high-level semantic features and low-level layer structure features for direct feature extraction. However, the lack of any parameters and an excessive reliance on the ResNet-50 network’s functionality make this architecture unsuitable for the extraction of sketch features. We intend to extract features that incorporate both global and local information, and the attention computation partially uses trainable parameters, which distinguishes our technique from EGFF and ExFuse in a key way. Similar semantic features of sketches and photos are aligned. We introduce a deep metric learning strategy to provide resilience and accurate retrieval. We utilize a modified form of self-attention as our attention component, since SIMAM [22], ATTENTION is computationally small compared to SE [23], CBAM [24], and ECA [25]. Contrary to self-attention, this technique creates the attention graph using data from the intermediate layer, which helps to instruct the model to concentrate on areas with high attention scores during the feature extraction stage. To improve the ability to extract features, we make certain unique alterations to the suggested method, which is not just a combination of EGFF, ExFuse, and attention. Significant performance gains are realized when comparing the proposed strategy to existing ones. To further investigate our AMFF module, an ablation study on feature fusion and feature layer selection was also conducted.

**Figure 1:**
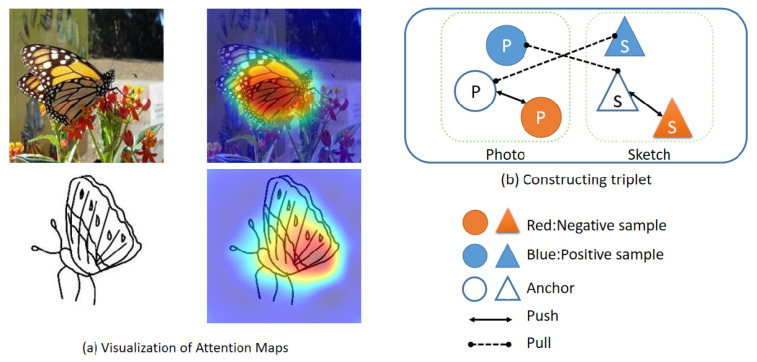
The illustration of our proposed model (**a**) Visualization of attention maps with different feature layers obtained using the ResNet-50 backbone (both sketches and photos are presented). (**b**) The triplet loss we construct can effectively reduce the domain gap.

To eliminate the large domain gap between sketches and photos, we propose a cross-modal triplet loss for ZS-SBIR that maps sketches and photos to a common semantic space, as shown in Figure 1. We use two basic deep metric learning (DML) [26,27,28], such as classification training and pairwise training. The former uses supervised information from the training set to construct a deep classifier, which motivates the model to create class-distinct deep embeddings for feature representations. We observe that previous work on ZS-SBIR has mainly used this learning approach [9,10]. The latter is a direct one-to-one comparison of attributes, consisting of an anchor image, a positive sample, and a negative sample in a feature triplet. The model tries to push the anchor away from the negative feature sample and bring the positive sample closer. The learned inter-class difference in the depth embedding is increased by adding a second margin to the feature triplet [29].

The study contributions are summarized as follows:

1. We proposed the Attention Map Feature Fusion (AMFF) network. AMFF combines the ResNet-50 network and the Attention network. With an excellent embedding ability, it can align similar semantic features of different modalities.

2. We proposed a domain-aware triplets to optimize the entire model to address the domain gap problem, which further narrows the modality gap with three types of pair-wise learning.

3. Extensive experimental results on three popular datasets demonstrate that our model outperforms the state-of-the-art by a significant margin.

## 2. Related Work

### 2.1. Deep Metric Learning

In recent years, the application of contrastive learning to self-supervised representation learning has made great progress. The application of contrastive learning in the unsupervised field has made the deep model achieve incredible performances, such as that of Prannay Khosla [30] of the famous American scientific research institution Google. The self-supervised and unsupervised domains are extended to the fully supervised domain, and a superior performance is obtained.

Self-supervised learning, which avoids the need to annotate large-scale data, is gaining popularity. As in a related work by Ashish Jaiswal et al. [31], it aims to embed enhanced versions of the same sample that are close to each other while trying to push away embeddings from different samples.

An efficient way to learn a distance metric between sample pairs isusing depth metric learning. Convolutional neural networks are used in deep metric learning to extract the samples’ deep features. In metric learning, there are three elements: positive samples and negative samples. We must choose an anchor point, increase the Euclidean distance between the anchor point and the negative sample, decrease the Euclidean distance with the positive sample, and employ multiple loss functions. For example, contrastive loss [32] and triplet loss [29,33] can only process two or three samples at a time, and only one negative sample is selected. Lifted-structure loss [34] can select all negatives at the same time. Use classification loss to explore the global relationship of each category, such as softmax loss [35].

### 2.2. Zero-Shot Sketch-Based Image Retrieval

ZS-SBIR is a zero-sample sketch retrieval task with a wide range of current practical applications. The analogy in the test phase is not visible in the training phase. For example, recent studies [3,12,15,17] explored representing images of different modalities in the same semantic space, thus enabling cross-modal sketch retrieval. However, the huge modality gap between sketches and photos poses a great difficulty for such exploration [16,36,37]. To address this domain gap, relatively new approaches [9,10] introduce semantic information to extract deep information from text semantics. However, this approach increases the computational effort, the added text semantic information is uncertain and some texts contain multiple semantics, which can easily lead the model to a bad local optimum. In contrast to these methods, our model uses the current, more popular contrast learning method to effectively address the modal domain divide. By reducing the distance between identical labels and increasing the distance between samples with different labels, our cross-modal triplet comparison method can fully exploit the relationship between sketches and photos to reduce the domain gap.

### 2.3. Feature Fusion

Feature fusion aims to combine different levels of semantic features. Many early works included related studies, such as fusing features from multiple intermediate layers. ExFuse [20] uses features from adjacent layers to guide feature extraction. SimAM [22] is based on an attention mechanism to fuse multi-layer features, which do not have any parameters. However, since this approach uses the features of each intermediate layer independently and does not connect the intermediate feature layers, this design does not much improve the final retrieval performance. Our goal is to obtain embeddings that contain both global and local features. We utilize global features to influence the extraction of local features, which is key to achieving accurate and stable retrieval. The recent work EGFF [19] uses energy strategy to achieve a combination of low-level structural features and high-level semantic features to guide the embedding of the most total features. However, because it does not involve any learnable parameters, his representation ability is insufficient for guiding the final feature embedding. Our method contains learnable parameters, which guide the final feature embedding.

## 3. Methodology

### 3.1. Problem Description

In the ZS-SBIR task, the dataset was split into two parts: training and validation. We trained the model in the training set and then tested our model in the validation set. The categories in the validation set are not available in the training set, where the training set is represented as $T^{\mathrm{seen}\;}=\left\{P^{\mathrm{seen}\;},S^{\mathrm{seen}\;}\right\}$. The test set is represented as $T^{\mathrm{unseen}\;}=\left\{P^{\mathrm{unseen}\;},S^{\mathrm{unseen}\;}\right\}$, where P represents the photo and S represents the sketch. We defined $P^{\mathrm{seen}\;}={\left\{\left(P_{j},y_{j}\right)|y_{j}\in C^{\mathrm{seen}\;}\right\}}_{j=1}^{N_{1}}$ and $S^{\mathrm{seen}\;}={\left\{\left(S_{i},y_{i}\right)|y_{i}\in C^{\mathrm{seen}\;}\right\}}_{i=1}^{N_{2}}$, where $y_{i}$ denotes the image classes, and $C^{\mathrm{seen}\;}$ denotes the existing categories. $N_{1}$ and $N_{2}$ represent the number of photos and sketches. *C* denotes the set of categories of images or sketches.

**Figure 2:**
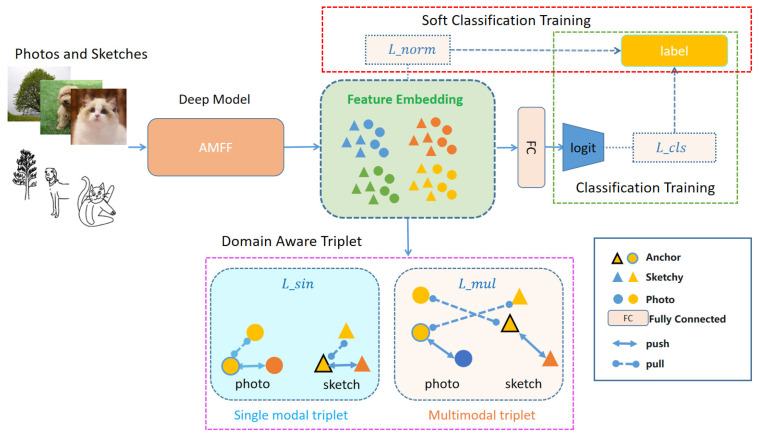
An overview of our proposed ZS-SBIR method.

We extracted sketches and images from the training set and use the corresponding labels as supervised information to direct the model to excellent feature embedding. In the testing phase, a sketch $Query\in S^{\mathrm{unseen}\;}$ that needs to be queried is given, where Query is not visible in the training set, and his label is $y\in C^{\mathrm{unseen}\;}$. If $C^{\mathrm{unseen}\;}$ denotes the category in the test set, such a category does not exist in the training set, and we considered a retrieval to be successful if the retrieved image label is the same as the sketch label that needs to be queried. An overview of our model is given in Figure 2.

### 3.2. Model Structure

We used the novel AMFF method to conduct feature embedding. We combined the embedding capability of the ResNet-50 network with the learning ability of the self-attention mechanism of the ATTENTION network to enable the model to learn useful features efficiently.

We used two forms of triplet (intra-modal triplet and hybrid triplet) and a gradient-based weighting module to complement the cross-modal triplet. The ternary loss largely reduced the domain gap, while we used cross-entropy loss to optimize the model and avoid bad local optima. Finally, the total embedding loss was calculated.

We used a single-stream network for feature extraction, mapping sketch and photo inputs into the same embedding space, as shown in Figure 2. The backbone network received the batch of carefully sampled sketches and photos to extract multilayer features, and then the AMFF module was applied for feature fusion of the different feature layers. Then, the model embedding was optimized using the basic method of deep metric learning (DML). The model embedding was optimized by the fully connected layers being converted to the same number of class dimensions for classification training, which will prevent the model from falling into a bad local optimum. This also enables further optimization of the model embedding.

#### 3.2.1. Attention Map Feature Fusion

**Figure 3:**
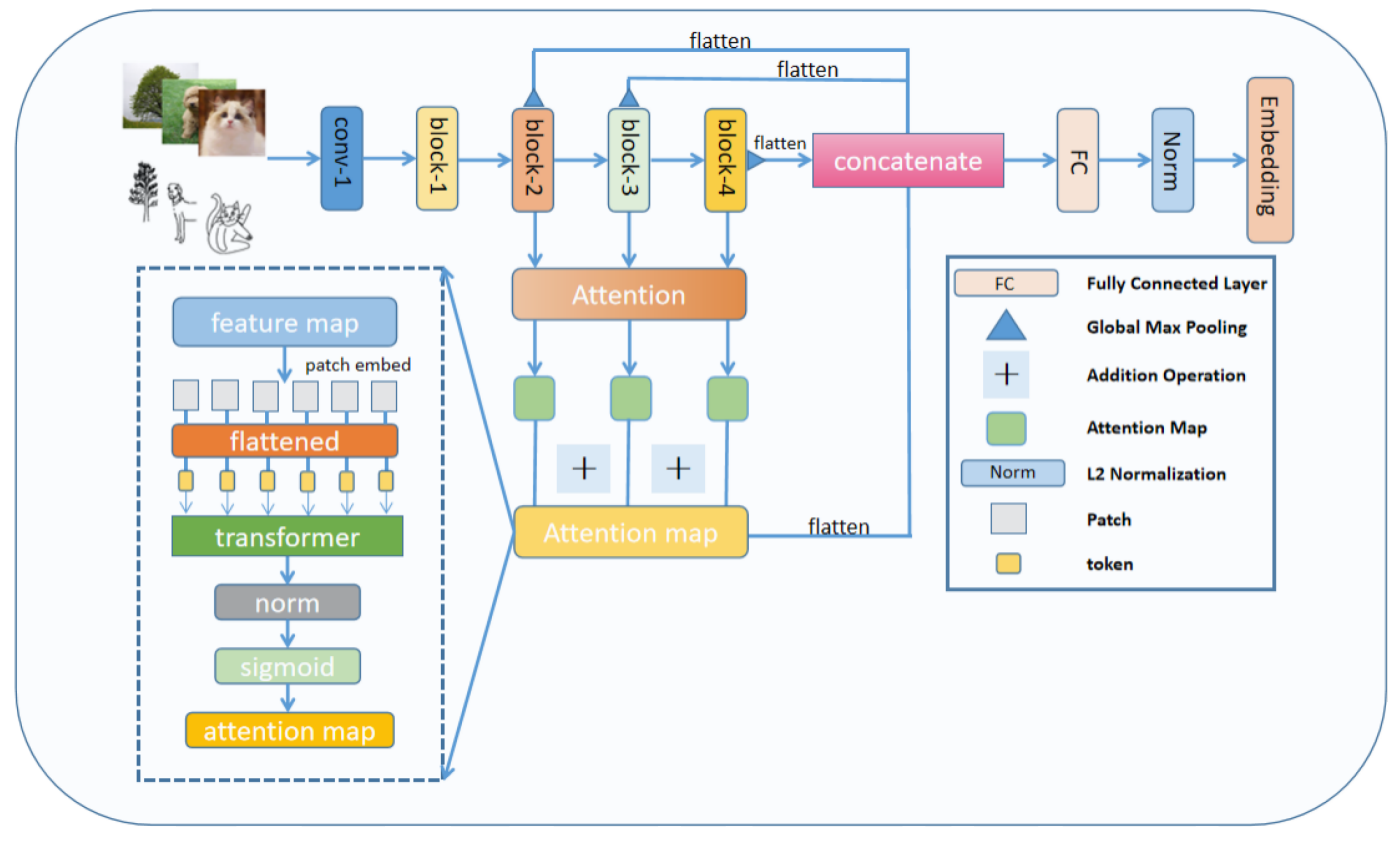
The general structure of the AMFF model.

Attention Map Feature Fusion (AMFF) combined the ResNet-50 [5] network and the attention network, respectively, recording the original features of different residual blocks and then using the output of the residual blocks as the input of the attention network. As shown in Figure 3, the attention network output the attention map, and then the output of the three different layers are summed to obtain the total attention map, and finally the new feature embedding was obtained by splicing with the output of the original feature layer. Finally, L2 normalization was employed to derive the retrieval task’s embedding dimension. It is worth noting that we added the attention maps of the three residual blocks and then stitched them together instead of using the original attention maps of the three residual blocks alone.

The feature extraction part is ResNet-50, where “Conv-1” denoted the first convolutional block of the backbone network and “blocks-1, 2, 3, and 4” denoted the residual blocks of the backbone network. The purpose of using the attention module to calculate the attention score of the residual blocks was to enhance and guide the final feature embedding using the semantic features of the intermediate layers, thus improving the retrieval performance of the whole model. The dashed box in the lower left corner was the data stream of the feature map.

#### 3.2.2. Attention

The AMFF module uses the transformer network of Vaswani et al. [21], and the dashed line in the lower left corner of Figure 3 shows the structure of the self attention. We used a convolution kernel size of 7 × 7 and a step size of 7. We linearly embedded each of the fixed-size patches from the feature map before feeding the resulting sequence of vectors to the transformer [38]. The sigmoid activation function was used after regularizing the outcome. The transformer’s attention mechanism enables the network to select which components to emphasize. The formulation of each Multi-Head Attention (MHA) layer is as follows:

(1) $$\mathrm{SHA}\left(k,q,v\right)=\mathrm{softmax}\left(\alpha qk^{T}\right)v.$$

(2) $$\begin{array}{c} MHA\left(k,q,v\right)=\left[SHA_{0}\left(kW_{0}^{k},qW_{0}^{q},vW_{0}^{v}\right),\ldots \right.\\ \left.SHA_{m}\left(kW_{m}^{k},qW_{m}^{q},vW_{m}^{v}\right)\right]W^{0} \end{array}.$$

where *k*, *q*, and *v* represent the Single-Headed Attention (SHA) module’s Key, Query, and Value inputs, respectively. The module determines how closely two feature pairs—Query and Key—are related, normalizes these scores, and then utilizes them as a projection matrix for Value features. The Multi-Head Attention (MHA) module combines the outputs of several single-heads and projects the outcomes to the lower dimension, where α is the scaling constant and *W* is the learnable weight matrix.

#### 3.2.3. Domain Aware Triplet

**Figure 4:**
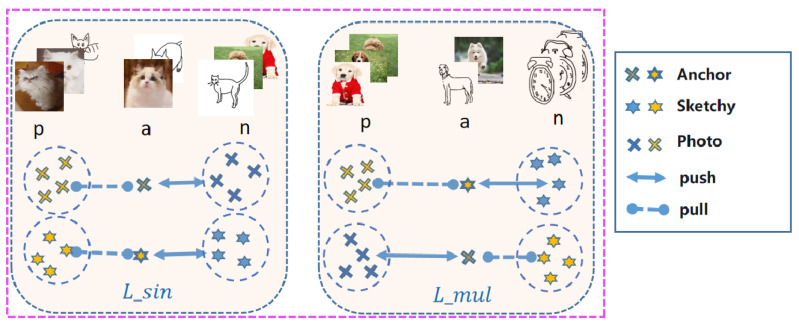
Our improved DAT triplet loss, where a represents the anchor point, p represents the positive sample, and n represents the negative sample.

We improved the domain-aware triplet(DAT), and based on the idea of self-supervision, we improved the ordinary triplet into $L_{sin}$ triplet and $L_{mul}$ triplet. It is worth noting that both positive and negative samples in $L_{sin}$ include two different modalities, as shown in Figure 4.

Single modal triplet. The basic method of metric learning consists of choosing an anchor point and its positive and negative samples, increasing the distance between the anchor point and the negative samples and decreasing the distance between the anchor point and the positive samples, where both the anchor point and the positive and negative samples are from the same modality ($m_{a}$=$m_{n}$=$m_{p}$) and the loss function is defined as follows:

(3) $$\begin{array}{cc} L_{sin} & =\left|E\left(x_{a}^{s},x_{i}^{s}\right)-E\left(x_{a}^{s},x_{j}^{s}\right)\right|\\ \; & +\left|E\left(x_{a}^{p},x_{i}^{p}\right)-E\left(x_{a}^{p},x_{j}^{p}\right)\right|+\gamma \cdot \end{array}$$

where *E* represents the Euclidean distance, a represents the anchor point, *s* represents the sketch, *p* represents the photo, $y_{a}$ = $y_{i}$ ≠ $y_{j}$.

Multimodal triplet. The main challenge in sketch retrieval is the domain gap, and learning discriminative feature embeddings is also crucial. The model was asked to discriminate whether the current input image is a sketch or a photo. Earlier works directly solved this problem using classification training, which does not make the model discriminative due to the huge domain gap problem, and the retrieval results of this method are very poor. Later on, generative adversarial networks were used to help the model learn discriminative properties. This method is difficult to train with a large number of parameters. Additionally, due to the significant domain difference between sketches and photos, generative adversarial networks may be used to degrade the expressiveness of feature embedding, further degrading SBIR performance. To address the modal discrepancy issue while maintaining inter-class variances, we designed hybrid triplet loss. An anchor photo, a cross-modal modal positive example image, and a homomodal negative example image make up the hybrid triplet. The corresponding loss function is written as:

(4) $$\begin{array}{cc} L_{mul} & =\left|E\left(x_{a}^{s},x_{i}^{p}\right)-E\left(x_{a}^{s},x_{j}^{s}\right)\right|\\ \; & +\left|E\left(x_{a}^{p},x_{i}^{s}\right)-E\left(x_{a}^{p},x_{j}^{p}\right)\right|+\gamma \end{array}.$$

The above loss function aims to reduce the domain gap of cross-modality $E\left(x_{a},x_{i}\right)$, where $x_{a}$ and $x_{i}$ are from different modality ($m_{a}$ ≠ $m_{i}$). It also raises the between-class disparity $E\left(x_{a},x_{j}\right)$, where $x_{a}$ and $x_{j}$ are from same modality ($m_{a}$ = $m_{i}$). Hybrid triplet loss suppresses the domain gap while maintaining between-class variation.

### 3.3. Training Approaches

#### 3.3.1. Embedding Learning

Normalized softmax loss [39], which is widely used in metric learning [25], has excellent embedding abilities, especially for the ZS-SBIR task, which guarantees that images from different modalities can be embedded in a common semantic space. We assign a learnable agent (represented as a vector) to each category, and our goal is to embed sketches and photos into the category agents and as far as possible from other agents. The objective function is (5).

(5) $$L_{norm}=-\log\left(\frac{\exp\left(\frac{x^{T}p_{y}}{t}\right)}{\sum_{z\in Z}\exp\left(\frac{x^{T}p_{z}}{t}\right)}\right).$$

where *Z* is the set of all agencies, $P_{y}$ is the agency of its category, and *t* is the temperature scale. as suggested in [39], we have set *t* = 0.05.

Classification training [40], the main purpose of this training was to stabilize the training process so that the model achieves a steady state in feature embeddings of different dimensions, and we used classification loss to store all classes of agents. Because pairwise training focuses on the internal relationship between each batch size, we needed classification training to stabilize the training of the model and avoid entering a bad local optimum. Classification training uses cross-entropy loss, whose formula is defined as follows:

(6) $$L_{cls}=-\sum_{i=1}^{N}\log\frac{\exp\left(\alpha_{y_{i}}^{⊤}f_{i}+\beta_{y_{i}}\right)}{\sum_{j\in C^{\mathrm{seen}\;}}\exp\left(\alpha_{j}^{⊤}f_{i}+\beta_{j}\right)}.$$

where α and β is the weight and bias of the classifier, and N denotes the number of samples in a training batch.

#### 3.3.2. Pairwise Training

Pairwise training [41], since the sketch and the photo in ZS-SBIR are from different modalities, we aimed to make the anchor point closer to his positive example image and further from his negative example image, i.e., the sketch has a smaller Euclidean distance from the image with the same label and a larger Euclidean distance from the image with different labels.

(7) $$E\left(x_{a}^{s},x_{i}^{p}\right)<E\left(x_{a}^{s},x_{j}^{p}\right),y_{a}=y_{i}\neq y_{j}.$$

where *E* denotes the Euclidean distance, $x_{a}$ denotes the anchor image, *y* denotes the category label, and *s* and *p* represent the sketch and photo, respectively.

To reduce the Euclidean distance between the anchor point and the positive example image, the distance between the anchor point and the negative example image should be increased. We propose a cross-modal method for reducing $E\left(x_{a}^{s},x_{i}^{p}\right)$ while increasing $E\left(x_{a}^{s},x_{j}^{p}\right)$. It is worth noting that the positive and negative example images of the anchor point are different from the modalities of the anchor point. They can also come from the same modality. To achieve the above goal, we introduced a cross-modal triplet where both sketches and photos are selected as anchor points. The cross-modal triplet loss is defined as:

(8) $$\begin{array}{cc} L_{\mathrm{cro}\;} & =\left|E\left(x_{a}^{s},x_{i}^{p}\right)-E\left(x_{a}^{s},x_{j}^{p}\right)\right|\\ & +\left|E\left(x_{a}^{p},x_{i}^{s}\right)-E\left(x_{a}^{p},x_{j}^{s}\right)\right|+\gamma \cdot \end{array}$$

where $x_{a}$ and $x_{j}$ are from various groups, whereas $x_{a}$ and $x_{i}$ are both from the same category. For improved categorization in the embedding space, a positive margin parameter was added.

#### 3.3.3. Objective and Optimization

The overall optimization function is (9). We seek the minimum value of *L*

(9) $$L=L_{norm}+\lambda \left(L_{cls}+L_{sin}+L_{mul}\right)\cdot$$

where λ is the hyperparameter that balances the performance of the model. More operation details are provided in the whole training procedure, which is presented in an Algorithm 1.
**Algorithm 1** Overall training procedure**Input:** training set $T^{\mathrm{seen}\;}=\left\{P^{\mathrm{seen}\;},S^{\mathrm{seen}\;}\right\}$; batch size N; hyperparameter of regularizer λ; **Parameter:** Model parameters $F\left(T^{\mathrm{seen}\;},\theta \right)$; classification layer $\left(W,b\right)$1:Freeze classification layer parameters2:**for** each iteration **do**3:      **for** each mini batch **do**4:            Calculate $L_{norm}$ Equation (5), $L_{cls}$ Equation (6), $L_{sin}$ Equation (3), $L_{mul}$ Equation (4)5:            Calculate $L=L_{norm}+\lambda \left(L_{cls}+L_{sin}+L_{mul}\right)$ Equation (9)6:            Update parameters with *∇L* by AdamW7:      **end for**8:**end for**9:**return** feature extractor $F\left(T^{\mathrm{seen}\;},\theta \right)$**Output:** Model parameters $F\left(T^{\mathrm{seen}\;},\theta \right)$;


## 4. Experiments

**Figure 5:**
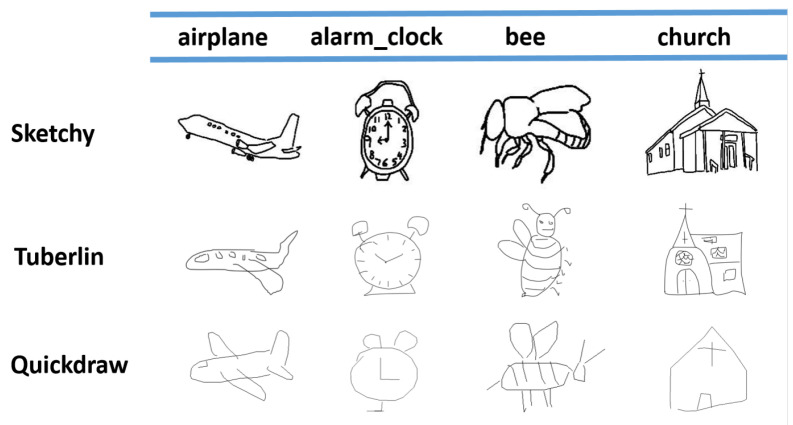
Qualitative comparison of Sketch datasets, with columns showing examples belonging to the same class.

In order to verify the efficiency of the method, we evaluated it using three popular datasets, namely Sketchy Extended [42], Tu-berlin Extended [43] and Quickdraw Extended [7]. As shown in Figure 5, the three datasets have increasing levels of abstraction.

### 4.1. Datasets

Sketchy Extended dataset consists of approximately 75,000 sketches and 12,500 images with a total of 125 different classes, and [44] randomly selected 25 classes as the validation set and 100 classes as the training set to train the model using the weights pre-trained in ImageNet [45]. However, the results show [3] that some categories in the validation set appear in the ImageNet pre-training data and, to avoid the influence of pre-training belonging, we selected 21 categories that do not appear in the ImageNet pre-training data for the validation set, and 104 categories of images for the training data. We conducted experiments in both the different validation categories of the same dataset. To distinguish the two, we named the former Sketchy_c100 and the latter Sketchy.

Tu-berlin Extended dataset is a challenging dataset with 250 different classes, and Liu et al. [14] extended this dataset with about 200,000 photos from the ImageNet dataset. The Tu-berlin Extended has only 20,000 sketches, so it is a very unbalanced dataset, with each sketch corresponding to ten photos. We followed the protocol to construct the training and validation sets [15], and we randomly selected 220 categories for the training set and 30 categories for the validation set (containing at least 400 images).

Quickdraw Extended dataset is a highly abstract dataset, containing a large number of sketches and photos. It has extremely low recognizability, which poses a greater challenge to the model. We followed a similar protocol to the partitioning proposed by Yelamarthi et al. [33], with a total of 110 different categories. We randomly selected 80 categories as the training set and 30 categories as the validation set.

The Sketchy, Tu-berlin, and Quickdraw datasets have increasing levels of abstraction. It is worth noting that, despite being the most abstract dataset, Quickdraw sketches can still be identified.

### 4.2. Evaluation Metrics

In the sketch retrieval (SBIR) task, precision and average precision are important measures of model performance, where precision (Prec@K) indicates the proportion of retrieved *K* photos (i.e., 100 or 200), of which the correct retrievals are among the total retrievals, and average precision (mAP@K), which calculates the average retrieval results for *K* photos.

(10) $$AP@K=\sum_{i=1}^{K}\frac{P@i\times \gamma (i)}{N}\cdot$$

*N* is the total number of relevant documents and γ(i) is 1 if the *i* ranked result is relevant; otherwise, it is 0. mAP@k is the mean AP@k of all queries.

### 4.3. Implementation Details

We used the Pytorch development framework with Ubuntu 20.04 LTS. Using an NVIDIA GeForce RTX 3090 GPU, we used AMFF pretrained on ImageNet as the backbone network, with learning rate set to $1\times e^{-5}$, learning rate decay set to $5\times e^{-4}$, batch size set to where the hyperparameters λ = 0.8, and the hyperparameter α is set to 0.2 for all ternary losses.

### 4.4. Comparing with the State-of-the-Arts

We compared our model with some excellent SBIR, ZSL, and ZS-SBIR techniques. Examples include the earlier results ZSIH [44] and Doodle2Search [7], both of which utilize a fairly simple framework that uses a single autoencoder or reconstruction loss to merge semantic information and visual features to obtain excellent embedding results. Recent works employ more complex frameworks, such as OCEAN [10] and SketchGCN [9]. To learn a shared semantic space, they combined generative adversarial networks with autoencoders. Another recent work, EGFF [19], combines low-level structural features and high-level semantic features to guide feature embedding, making the final feature embedding more linearly separable. Our approach was compared with two models that use a deep metric learning (DML) approach, e.g., MATHM [41], DSN [40], both of which use the classical depth metric learning approach for model optimization. To be fair, some of our comparison experiments were performed using the same setup, and some of the earlier experimental results are cited in other papers.

In most experiments, we showed better performance than other models, and we even outperformed many approaches using text semantic-assisted models. We also compared different embedding dimensions. We used an embedding dimension of 512 dimensions, and we also conducted related experiments in 64 dimensions, with both showing excellent performance. The comparison results in the Sketchy and Tu-berlin datasets are shown in Table 1. Table 2 shows the comparison results in the Quickdraw dataset.

### 4.5. Qualitative Results

**Figure 6:**
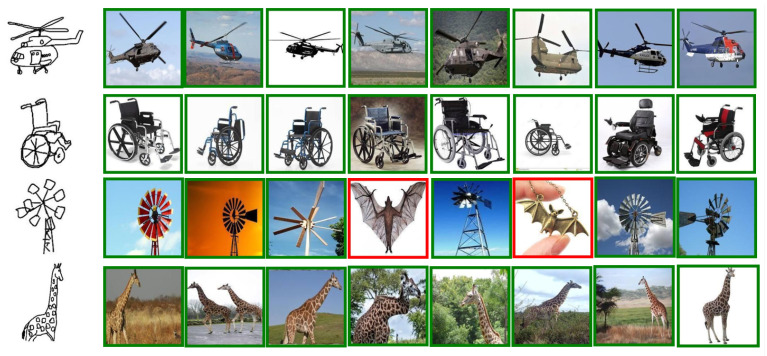
Top-8 results on the Sketchy dataset.

**Figure 7:**
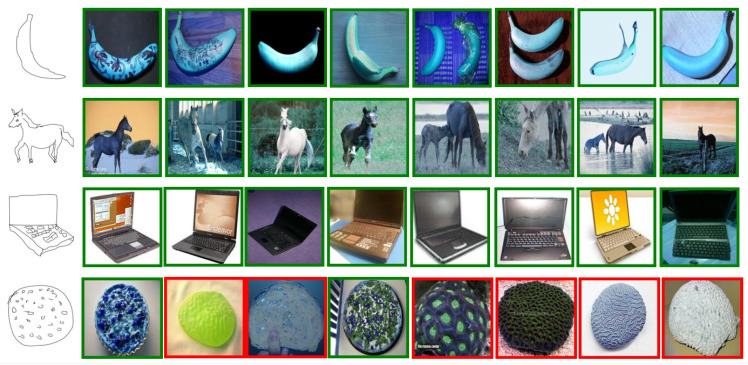
Top-8 retrieved by our model on Tu-berlin Extended datasets.

**Figure 8:**
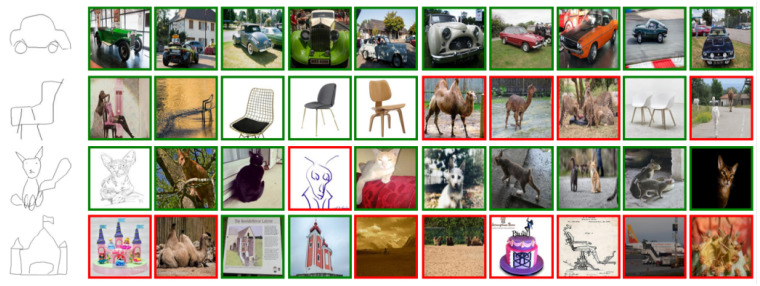
The top 10 ZS-SBIR results were retrieved from the Quickdraw Extended dataset.

We have conducted relevant validation experiments on three different datasets, and we show part of the results, including successful or failed cases, among which we show the top 8 results for the Sketchy dataset and the Tu-berlin dataset, and top 10 results for the retrieval of the Quickdraw dataset. Figure 6 and Figure 7 show the retrieval results for Sketchy and Tu-berlin, respectively, and Figure 8 shows the retrieval results of Quickdraw. As can be seen from the figures, our method can retrieve exactly the right results when the retrieved target has obvious appearance characteristics and vice versa. If the retrieved sketch does not have a clear appearance, many wrong results may be retrieved.

As shown in Figure 6, green borders indicate correct retrieval results and incorrect retrieval results are indicated by red boxes. As shown in the figure, rows 1, 2, and 4 are completely correct, and the third row shows some incorrect results.

Correct results are shown with green borders Figure 7, while incorrect results are shown with red borders. The top three rows are all correct, and the fourth row is partially correct.

Correct results are shown with green borders Figure 8, while incorrect results are shown with red borders. The first row is correct in all cases. The second, third, and fourth rows have varying degrees of errors, with the fourth row showing the most incorrect results.

The qualitative results are shown in Figure 6, Figure 7 and Figure 8. We created a visualization of four different sketches that were randomly selected from each dataset. The visualization results illustrate the effectiveness of our proposed method. For example, Figure 6, the Sketchy Extended dataset, retrieved the correct photos for “banana”, “horse”, and laptop in rows 1, 2, and 3. For some difficult cases in Figure 7, such as the sketch “pizza” in row 4 of the Tu-berlin Extended dataset, it is very difficult to distinguish “pizza” because its appearance is not obvious and there are many similar photos. For example, as the level of sketch abstraction increases, the query sketch will be more ambiguous and it will be increasingly difficult to obtain the correct retrieved photo. For example, in Figure 8, the second and fourth rows have very serious retrieval errors because many photos have very similar appearance features, and the more abstract the query sketch is, the more difficult it is to retrieve. Therefore, for more challenging and abstract cases, In Figure 8, row 2, “chair,” the 6th photo retrieved belongs to “camel,” and we think this failure is because the retrieved error results are visually similar to the given query sketch. They share a common search space.

### 4.6. Ablation Studies

**Figure 9:**
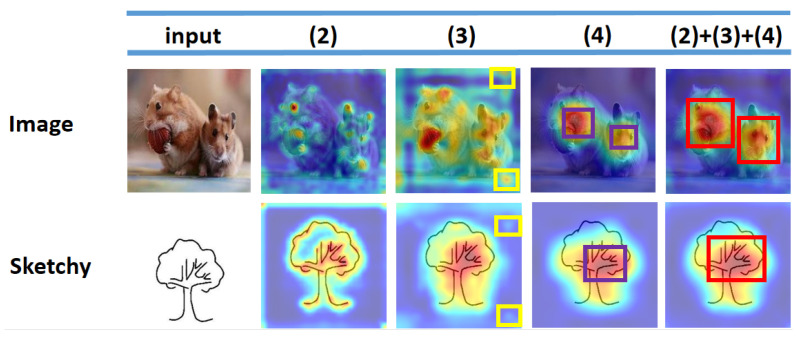
We visualize the attention map of our proposed backbone network. The model in the lower-level features (i.e., levels 2 and 3) is not fully focused and has numerous spurious signals (yellow box), while the higher-level features (feature level 4) focus only on the distinguished regions and ignore the local regions (red box).

We also explored different combinations of feature layers on the Sketchy Extended dataset, and the results are shown in Table 3. The results show that the best results can be obtained by combining higher-level semantic features with lower-level semantic features (using features from levels 2, 3, and 4). The attention map based on ResNet-50 for different feature layers is shown in Figure 9. We can see that the model in the lower-level features (i.e., levels 2 and 3) is not fully focused and has numerous spurious signals, as shown in the yellow rectangular box in the figure, while the higher-level features (feature level 4) focus only on the distinguished regions and ignore the local regions (red box). Our proposed method pays more attention to detail by combining features at different levels and, with an increasing range of attention, more useful features can be extracted from sketches or photos.

Figure 9 shows the results obtained by fusing different layers. The results show that optimal results are achieved when fusing layers 2, 3, and 4.

To verify the validity and importance of each different component, we first traiedn a shared semantic space containing only classification losses. Then, we optimized the model with each of the three different triplet loss functions. The results show that each type of triplet loss improves the performance of the model by different magnitudes. As seen in Table 3, the intra-modal triplet loss and cross-modal triplet loss do not improve mAP and Prec significantly, while the mixed triplet loss outperforms the other loss functions and is close to the optimal result. This implies that the mixed triad loss is more important than the cross-modal triad loss in our model. However, using these three loss functions separately does not lead to the best performance. The last row uses all loss functions to achieve the best performance, and it is worth noting that the best performance was achieved using only the mixed triplet prec@100 in the Sketchy dataset.

Due to the particularity of our method, the complexity of our method is high. In order to evaluate the relationship between revenue and model complexity, we compared several works in the last two years on the Tuberlin dataset. We used an RTX 3090 GPU with a batch size of 64. As can be seen from the results, our method does not sacrifice much training and inference time. In future research, we will reduce the model’s complexity to better balance the relationship between complexity and benefit, as shown in Table 4.

**Figure 10:**
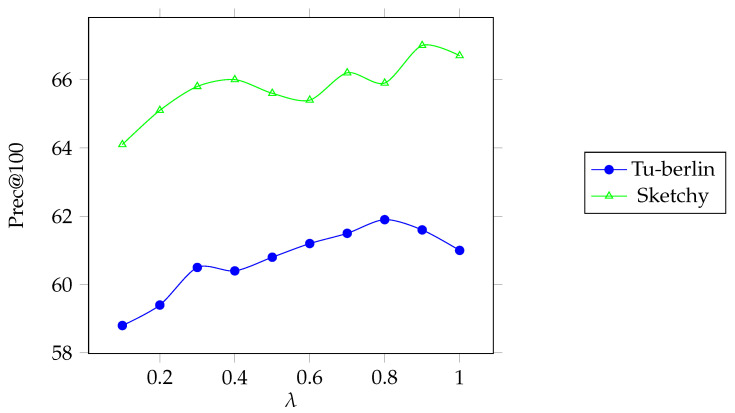
Parameter sensitivity analysis. The blue part represents the results on the Tu-berlin dataset, and the green part represents the results on the Sketchy dataset.

We introduced the parameter λ in order to balance the performance of the model. We studied the impact of different λ on performance. We conducted experiments on the Sketchy and Tu-berlin. We took a different λ value, and the Figure 10 showed that the best performance was obtained when λ ∈ (0.7, 1).

In order to verify the validity of AMFF, we used the same method for training and compare several major embedding networks. The results show Table 5 that our AMFF is effective.

## 5. Limitation

Our method had an excellent performance on the Sketch and Tu-berlin datasets but did not perform well on the Quickdraw dataset. We speculate that this dataset is too abstract, which causes the first residual block of the ResNet-50 network to extract meaningless features, thus leading to the failure of the attention network, which we will explore in subsequent work. In addition, since our introduction of the attention network leads to an increase in the number of model parameters and computational complexity, we will explore simplifying the model to obtain a better performance in our subsequent work to address this problem.

## 6. Conclusions

In this paper, we propose a useful network for ZS-SBIR. First, we propose the AMFF model as a backbone network. This approach enhances the embedding capability of the model by combining the efficiency of the ResNet-50 network with the representation capability of the attention network. Second, we used classification loss to solve the stability problem during training and avoid bad local optima, and finally, we constructed a modality-aware triplet with enough positive and negative samples to smooth the domain gap. We conducted extensive experiments on three popular datasets, namely, Sketchy, Tu-berlin, and Quickdraw. Experiments show that our method achieved the performance of the current best methods.

## Figures and Tables

**Table 1:** Comparison of retrieval performance with existing mainstream methods (where best performance is shown in bold).

Task	Methods	Dimention	Sketchy_c100		Sketchy		Tu-Berlin	
			mAP@all	Prce@100	mAP@200	Prec@200	mAP@all	Prce@100
SBIR	GN Triplet (2016) [42]	1024	20.4	29.6	-	-	17.5	25.3
	$DSH_{b}$ (2017) [14]	64	17.1	23.1	-	-	12.9	18.9
ZSL	SAE (2017) [46]	300	21.6	29.3	-	-	16.7	22.1
	FRWGAN (2018) [44]	512	12.7	16.9	-	-	11.0	15.7
ZS-SBIR	Doodle2Search (2019) [7]	4096	-	-	46.1	37.0	10.9	-
	Sake (2019) [8]	512	-	-	49.7	59.8	47.5	59.9
	SketchyGCN (2020) [9]	1024	38.2	53.8	-	-	32.4	50.5
	OCEAN (2020) [10]	512	46.2	59.0		-	33.3	46.7
	PCMSN (2020) [12]	64	52.3	61.6	-	-	42.4	51.7
	SBTKNet (2021) [18]	512	55.2	69.7	50.2	59.6	48.0	60.8
	DSN (2021) [40]	512	58.1	70.0	-	-	49.3	60.7
	NAVE (2021) [17]	512	61.3	72.5	-	-	48.4	59.1
	MATHM (2021) [41]	512	62.9	73.8	48.5	58.1	46.1	59.8
	EGFF (2022) [19]	512	62.3	75.5	51.7	61.2	46.2	60.4
	BDA-SketRet (2022) [47]	64	-	-	43.7	51.4	37.4	50.4
	FFMLN (ours)	64	55.9	67.8	46.1	56.2	44.0	54.4
	FFMLN (ours)	512	**65.6**	**77.0**	**53.6**	**62.4**	**49.3**	**61.9**

**Table 2:** For the ZS-SBIR task, the performance of our proposed method on the Quickdraw Extended dataset; the best performance is shown in bold.

Methods	Dimention	Quickdraw		
		mAP@all	mAP@200	P@200
CVAE (2018) [3]	4096	0.30	-	0.30
Doodle2Search (2019) [7]	4096	7.52	9.01	6.75
SBTKNet (2021) [18]	512	11.9	-	16.7
FFMLN (ours)	64	26.7	29.3	39.7
FFMLN (ours)	512	**28.8**	**34.5**	**45.1**

**Table 3:** The results obtained by using different optimization functions (embedding dimension is 512).

Loss Function	Sketchy_c100		Tu-Berlin	
	map@all	prec@100	map@all	prec@100
$L_{norm}$	61.01	75.23	44.73	59.47
$L_{norm}$ + $L_{cls}$	61.96	75.55	46.57	60.39
$L_{norm}$ + $L_{cls}$ + $L_{in}$	64.22	76.22	47.91	60.85
$L_{norm}$ + $L_{cls}$ + $L_{hyb}$	65.52	**77.11**	48.92	61.53
FFMLN (ours)	**65.63**	77.05	**49.30**	**61.90**

**Table 4:** Assess the relationship between yield and model complexity. Inference time represents the number of images per second that can be processed in our operating environment.

Method	Parameter	Convergence	Training Time	Inference Time	mAP@all
EGFF (2022) [19]	56.19 M	10	119.8 min	286	46.2
DSN (2021) [40]	54.36 M	16	346.1 min	-	49.3
Ours	290.1 M	6	81 min	278	49.3

**Table 5:** Overview of Feature Embedding Capabilities.

Task	Embedding Method	Sketchy_c100	Tu-Berlin
		Prce@100	mAP@all
ZS-SBIR	VGG-16	59.8	36.9
	CSE_ResNet-50	73.8	46.1
	EGFF	75.5	47.2
	Siamese CNN	73.1	46.5
	AMFF (ours)	**77.0**	**49.3**

## Data Availability

Our code and related datasets are publicly available at https://github.com/haizhu12/ammln.git.
